# Crystal structure of apo human spermidine synthase reveals dynamic rearrangement at the active site

**DOI:** 10.1063/4.0001199

**Published:** 2026-01-16

**Authors:** Omowumi O. Fagbohun, Molly A. Canfield, Jonathan A. Clinger

**Affiliations:** Department of Chemistry & Biochemistry, Baylor University, One Bear Place #97028, Waco, Texas 76798-7028, USA

## Abstract

Polyamines are polycations involved in both differentiation and proliferation of cells. Highly conserved polyamine biosynthetic enzymes are involved in the synthesis of polyamines. Spermidine synthase (SPDS) is an important enzyme in the polyamine biosynthetic pathway, and it is an aminopropyl-transferase that catalyzes the synthesis of the polyamine, spermidine, from putrescine and decarboxylated S-adenosine methionine. Spermidine has a variety of biological roles, including the formation of eIF5A, regulating autophagy, and stabilizing DNA and RNA. While there are numerous structures of human SPDS in complex with its substrates, products, or inhibitors, and numerous apo structures from various species, there is no structure of the apo form of human SPDS reported to date. In this study, the crystal structure of apo human SPDS was determined at 1.95 Å resolution. Comparison of the inherently flexible gatekeeping loop in the apo human structure with apo homologues revealed species-specific differences in loop conformation, indicating dynamics. Significant conformational change was observed in active site residues that are involved in catalysis when the apo human structure was compared to human ligand-bound complexes. These findings provide structural insights into the conformational dynamics and ligand-binding properties of spermidine synthase.

## INTRODUCTION

Polyamines are prevalent polycations and crucial components of cells, with putrescine, spermine, and spermidine being the most common.^1–5^ They are involved in the growth and development of cells.^6–8^ The charge distribution of polyamines enables them to neutralize and provide stability to nucleic acids. Polyamines play a number of roles in supporting cell growth and, in certain situations, survival, including interacting with nucleic acids, preserving chromatin structure, controlling the expression of certain genes, regulating ion channels, stabilizing membrane, and scavenging free radicals.^1,9–13^ Aminopropyltransferase-catalyzed reactions generate polyamines in eukaryotes and in numerous prokaryotes.^6^ These aminopropyl-transferases are intriguing targets for the advancement of antiproliferative therapies because of the close link between polyamine production and cell growth. The aminopropyl-transferase family includes ornithine decarboxylase, spermidine synthase, and spermine synthase. The synthesis of spermidine and spermine is dependent on the aminopropyl donor known as decarboxylated S-adenosyl methionine, which is synthesized by the enzyme S-adenosyl methionine decarboxylase from S-adenosyl methionine.^14–18^

The metabolism and levels of polyamines are often poorly regulated in cancer.^1,19^ For instance, proliferation in breast cancer, colorectal cancer, and cell death are significantly influenced by polyamine metabolism.^6^ In most cancer types, elevated levels of polyamines and high activity of polyamine enzyme have been found.^20^ The dysregulation of polyamine metabolism observed in cancer is mostly due to the overexpression of the enzymes responsible for polyamine biosynthesis.^6,21^ This results in an increase in the level of polyamines, which are essential for the growth of tumors and malignant transformation.^21^ Thus, the pathway for polyamine biosynthesis is an essential target for the development of therapeutics.

Spermidine synthase (SPDS) catalyzes the transfer of the aminopropyl group from decarboxylated S-adenosylmethionine (dcAdoMet), a universal cofactor, to putrescine (PUT), forming spermidine (SPD) and a by-product, 5-deoxy-5-methylthioadenosine (MTA).^1,22^ Many identified SPDSs, including the human SPDS, are highly selective for putrescine and do not utilize spermidine as an alternative substrate, even though the reactions produced by the two latter enzymes in the pathway are extremely similar.^5,15,16,23^ This restriction is caused by the residue Trp28, which closes off the substrate-binding cavity, thus preventing the binding of longer substrates in the active site of SPDS.^5,22^ Also, putrescine is not used by spermine synthase as a substrate.^5,23^ Other aminopropyl-transferases, like those found in acute thermophiles that produce a range of polyamines absent in mammals, tend to be less selective.^5,15,16,24^ Spermidine is one of the main polyamines involved in growth, and it also acts as a precursor to hypusine, which is necessary for the functioning of the elF5A initiation factor in eukaryotes. eIF5A plays an important role in protein synthesis; it is involved in initiation, elongation, and termination. It serves as a source of the aminobutyl group needed for the amino acid lysine of the translation factor to undergo posttranslational modification.^15,22,23,25–28^ Additionally, spermidine plays a role in a number of biological functions, such as promoting autophagy, inhibiting synthesis of nitric oxide, and controlling membrane potential.^29^

The human SPDS exists as a dimer with a separate substrate-binding site in the two chains. Each chain consists of the N-terminal, central core, and C-terminal domains. The dimer interface of human SPDS is formed by the N- and C-terminal domains. The binding of spermidine, putrescine, dcAdoMet, and MTA has been used to reveal the active site of human SPDS.^4,5^ It is composed of conserved residues from the SPDS family, and it is between the central core domain and the N-terminal domain. The active site is made up of both the putrescine binding pocket and the aminopropyl donor binding pocket. Both spermine synthase and SPDS use similar catalytic mechanisms. The putrescine amine is deprotonated by Asp173 and then performs a nucleophilic attack on the aminopropyl group's carbon linked to the sulfonium center of dcAdoMet, leading to the formation of spermidine and MTA. The conserved Asp173, together with the backbone CO group of amino acid residue Ser174 and the OH groups of conserved amino acid residues Tyr241 and Tyr79, stabilizes and positions the nucleophilic amine of putrescine. The formation of spermidine and MTA is completed by the transfer of an electron pair to the sulfur atom. An essential component of this reaction is the positively charged sulfur atom.^5,15^ There is also a flexible gatekeeping loop that covers the active site, which is disordered in the apo structure but stabilized upon the binding of the substrate.^15,30^ This loop in *Thermotoga maritima* in complex with S-adenosyl-1,8-diamino-3-thiooctane (AdoDATO) was revealed to be ordered and completely envelops the active site.^4^

There are many deposited structures of SPDS from different organisms, and they include apo structures from *Thermotoga maritima* (1INL),^4^ *Trypanosoma cruzi* (3BWB),^31^ *Bacillus subtilis* (1IY9),^32^ *Kluyveromyces lactis* (8IYI),^29^ *Plasmodium falciparum* (2PSS),^33^ and *Arabidopsis thaliana* (1XJ5).^34^ There are also structures of human SPDS in complex with MTA (2O05), MTA and putrescine (2O06), MTA and spermidine (2O07), dcAdoMet (2O0L),^5^ and decarboxylated S-adenosylhomocysteine (dcSAH) (3RW9).^15^ However, there is no reported structure of the apo human SPDS. Here, we determined the crystal structure of the apo human SPDS at 1.95 Å resolution. This allows for comparison of the apo human SPDS with other homologous structures and ligand-bound human structures. Differences in the active site residues between the apo state and the ligand-bound states were observed, particularly the catalytically important residues, Asp173 and Ser174.

## METHODS

### Expression and purification

A modified pET-27b (+) vector containing the human SPDS gene with TEV cleavage site downstream of 6× Histag at the N-terminus was purchased from GenScript. The plasmid DNA was transformed into *Escherichia coli* BL21 competent cells purchased from New England Biolabs. Human SPDS (Accession number: P19623) was produced using chemically competent *Escherichia coli* BL21 (DE3) cells and the cells were grown in 2×YT media at 37 °C to OD600 of 0.6–0.8 with 50 *μ*g/ml of kanamycin. Expression was induced using 1 mM isopropyl β-D-1-thiogalactopyranoside and incubated at 15 °C overnight. The cell culture was centrifuged for 45 min at 6000 rpm. Pellets were collected and resuspended in a buffer containing 5 mM imidazole, 100 mM HEPES (2-[4-(2-hydroxyethyl) piperazin-1-yl] ethane sulfonic acid), and 250 mM sodium chloride. A sonicator was used to lyse the cells for 20 min (“30 s” on and “20 s” off pulses), after which the lysate was centrifuged using a high-speed centrifuge at 21 000 rpm for 30 min. Ni^2+^ was used to charge the Ni-NTA column (HisTrap HP, Cytivia), which was used for the purification of the protein. The running buffer contained 5 mM imidazole, 100 mM HEPES pH 7.5, and 250 mM sodium chloride, and the elution buffer was identical, except that the concentration of imidazole was increased to 500 mM. Size exclusion chromatography (HiLoad 16/600 Superdex 200PG, Cytivia column) was used for further purification of the protein, and the buffer contained 25 mM HEPES pH 7.5 and 200 mM sodium chloride.

### Crystallization

Employing the sitting drop vapor diffusion approach, crystallization of human SPDS protein was done on a Swissci 96-well 2-drop plate with the NT8 crystallization robot. The crystallization conditions for human SPDS contained 0.05–0.225 M sodium sulfate, 0.1 M Bis-Tris buffer of pH 5.5, and 6.5%–23% polyethylene glycol 3350. The top wells contained a 1:1 v/v of 10 mg/ml of SPDS to the reservoir solution. Crystals grew after 24–28 h at room temperature, and crystal extraction was done in a cold room at 4 °C. The crystals were cryoprotected with mother liquor containing 10% glycerol, looped, and plunged into liquid nitrogen.

### Data collection, processing, and refinement

X-ray diffraction data were collected at the Cornell High Energy Synchrotron Source (CHESS), Cornell University, New York. The diffraction data were processed using the automated FastDP pipeline provided by CHESS for spot-finding and indexing, which incorporates XDS, CCP4, CCTBX.^35–38^ POINTLESS was used for space group assignment and AIMLESS were used to further scale and merge data.^39^ The output file from AIMLESS was used to run Xtriage on Phenix (version 1.21.2-5419-000) to ensure there were no warnings present.^40^ Phenix refinement was used to adjust the coordinates of atoms in the structure using the human SPDS MTA+PUT bound structure (PDB 2O06) as the starting structure, which has the same space group and a similar unit cell parameters.^5^ Manual model building was done in COOT (version 0.9.8.95).^41^ The data collected were processed to a resolution of 1.95 Å. Table I provides a summary of the statistics for data collection.

**TABLE I.** Data collection, processing, and refinement statistics.

PDB ID	9Q41
Wavelength (Å)	0.8857
Resolution range (Å)	29.64–1.95 (2–1.95)
Space group	C2221
Unit cell	
a, b, c (Å)	118.568 133.704 82.369
*α*, *β*, *γ* (°)	90 90 90
Total reflections	330 416 (23 275)
Unique reflections	47 994 (3 452)
Multiplicity	6.9 (7.0)
Completeness (%)	100.0 (100.0)
Mean I/sigma (I)	4.8 (1.1)
Wilson B-factor	27.78
R-merge	0.237 (1.795)
R-meas	0.256 (1.94)
R-pim	0.097 (0.73)
CC1/2	0.978 (0.457)
Reflections used in refinement	47 994 (3451)
Reflections used for R-free	1 999 (140)
R-work	0.164 5 (0.258 1)
R-free	0.213 1 (0.290 8)
Number of non-hydrogen atoms	4 889
macromolecules	4 429
ligands	0
solvent	460
Protein residues	556
RMS [bonds (Å)]	0.007
RMS [angles (°)]	0.85
Ramachandran favored (%)	97.45
Ramachandran allowed (%)	2.55
Ramachandran outliers (%)	0
Rotamer outliers (%)	0
Clashscore	1.93
Average B-factor (Å^2^)	32.67
macromolecules	32.19
ligands	0
solvent	37.25

MTA and PUT were placed in the active site of apo SPDS and re-refined to demonstrate poor fit to experimental data. The B-factors of the ligands were set to the B-factor of the surrounding residues and refined again. The MTA molecule in chain A was flipped by Phenix to find a better fit, but the fit remained poor (Fig. S6). Homologous SPDS structures were downloaded from RCSB.org, protein sequence alignment was done using ESPript 3.0 and PyMOL was used for the generation of all structure figures.^42,43^ Additional analysis by Ringer was used to compare the side chain rotameric conformation of apo vs MTA+PUT bound SPDS structure.^44^ The structure of apo human SPDS was deposited in the Protein Data Bank with PDB ID 9Q41. Diffraction images were deposited on SBGrid Data Bank with dataset number 1194 (doi:10.15785/SBGRID/1194).

## RESULTS/DISCUSSION

The crystallization of apo human SPDS generated needlelike crystals up to 200 *μ*m long. During the initial data collection trials, the crystals failed to diffract under the normal cryo-handling procedure, resulting in weak to no diffraction spots. Upon closer observation, we realized that these crystals dehydrated while looping and transferring to liquid nitrogen even though they were cryo-protected. Crystal dehydration, especially uncontrolled, can disrupt crystal order and crystal lattice, increase mosaicity, and collapse unit cell.^45^ Subsequent crystal extraction was done in a cold room, which slowed dehydration due to evaporation. Crystals prepared this way diffracted to a resolution of 1.95 Å (Table I). The morphology of the crystals that were looped at room temperature, which did not diffract, had a bent conformation after mounting. This is likely due to the uneven loss of water that produces mechanical stress manifesting as visible bending (Fig. S1). This suggests that better diffracting crystals resulted from the improved hydration of crystals that were looped in the cold room (Fig. S2). Morphologies of the diffracting and non-diffracting crystals before looping were the same; however, the outcomes differed greatly, underscoring the hydration sensitivity of this crystal form.

The structure of apo human SPDS exists as a dimer [Fig. S3(a)]. Each dimer consists of the N-terminal, central, and C-terminal domains [Fig. S3(b)]. The N-terminal domain is made up of six beta strands of residues 14-72, with the first two beta strands forming a β-hairpin and the other four forming an antiparallel beta sheet. The central domain is the largest domain, and it is the catalytic core domain made up of seven alpha helices and seven beta strands that form a Rossmann-like fold. It consists of residues 73-267. The C-terminal domain is the smallest domain, made up of three alpha helices from residues 268-302. The N-terminal and the C-terminal domains form the surface for dimerization, with the second beta strand of both chains in proximity, leading to the formation of a combined four beta strands from both chains into a beta sheet.

**FIG. 1.**
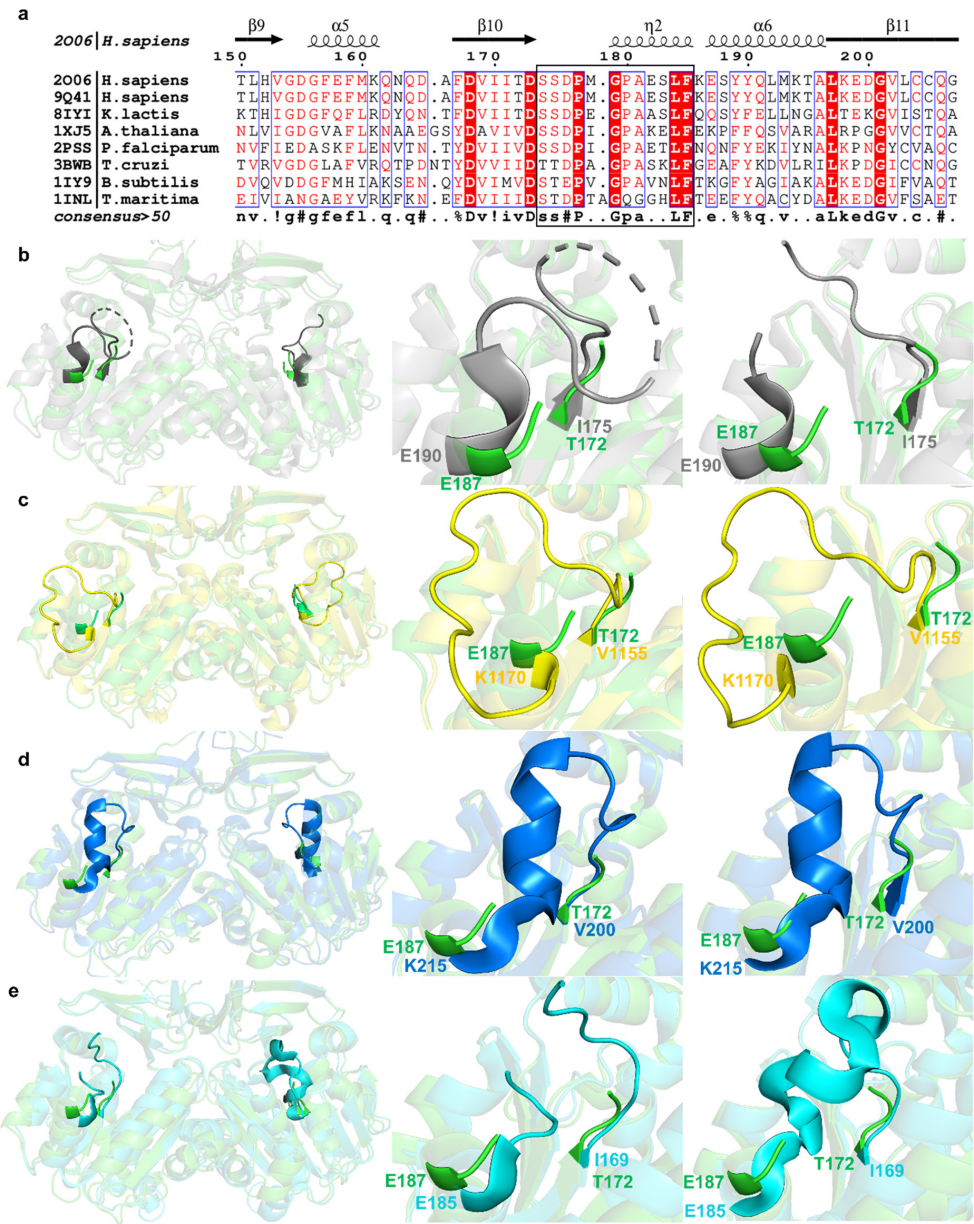
(a) Protein sequence alignment of SPDS from different organisms generated using ESPript 3.0. The black box represents the gatekeeping loop region. Description is the same as Fig. S4. (b)–(e) Gatekeeping loop comparison of apo human SPDS structure with other apo SPDS structures from homologous species. Superposition of apo human SPDS (green, chains A and B) with (b) *Trypanosoma cruzi* (3BWB) in gray (chain A and chain B), (c) *Bacillus subtilis* (1IY9) in yellow (chains B and C), (d) *Arabidopsis thaliana* (1XJ5) in blue (chains A and D), and (e) *Thermotoga maritima* (PDB 1INL) in cyan (chains B and C).

The gatekeeping loop of apo SPDS is flexible and exhibits a high degree of disorder. To explore the differences in the gatekeeping loop, the apo form of human SPDS was aligned and superimposed with various apo SPDS structures from other species (Figs. 1, S4, and S5). The gatekeeping loop corresponds to residues S174 to E182 in human SPDS ligand-bound structures;^5^ however, the disorder extends beyond E182 to F185 in apo human structure (Fig. 1). Density for this loop in chains A and B (residues 175–185, 11 residues) is absent in the apo human SPDS structure, suggesting high flexibility and disorder in the absence of ligand. This is in agreement with the established function of the loop in active site regulation and ligand gating.^5^ In the structure of *Trypanosoma cruzi* (PDB 3BWB), the gatekeeping loop showed the absence of three residues in chain A and the absence of four residues in chain B [Fig. 1(b)]. In the *Kluyveromyces lactis* apo SPDS structure (PDB 8IYI), there is only an absence of two residues in chain A and complete residues in chain B in the corresponding gatekeeping loop, indicating partial disorder [Fig. S5(a)]. This loop is complete with no absence of residues in the four chains of *Bacillus subtilis* [Fig. 1(c)], a tetramer (PDB 1IY9), the same as in the structure of *Arabidopsis thaliana* [Fig. 1(d)], also a tetramer (PDB 1XJ5). However, this loop in *Bacillus subtilis* is in a different position compared to the loop in *Kluyveromyces lactis* and *Arabidopsis thaliana* [Fig. S5(b)]. The loop is bent off from the active site, with large deviation from homologous structures. This loop in *Kluyveromyces lactis* is also in a different conformation than *Arabidopsis thaliana*, with an inward conformation observed in *Arabidopsis thaliana*.

In the apo structure from *Plasmodium falciparum* (PDB 2PSS), the gatekeeping loop is also very flexible with the absence of 12 residues, which is comparable to the apo human SPDS [Fig. S5(c)]. The structure of *Thermotoga maritima* is a tetramer with two subunits of the tetramer dimerizing at the N-terminal and the C-terminal domains, just as in the apo human structure. The first two beta strands that form a β-hairpin at the N-terminal domains of the four subunits form a beta barrel, providing the surface for the formation of the tetramer (PDB 1INL). Each dimer of the tetramer superimposes well with the apo human structure. The gatekeeping loop of *Thermotoga maritima* is missing 10 residues in chain A and four residues in chain B. Chain C is complete, while chain D is missing 11 residues. Chains A and D dimer appear to be highly disordered [Fig. S5(d)], similar to apo human SPDS, and chains B and C dimer show less disorder in this loop region [Fig. 1(e)]. The gatekeeping loop in *Kluyveromyces lactis,* described in an open position by Kim and Chang,^29^ adopts the most outward position compared to other homologues, except the gatekeeping loop in *Bacillus subtilis,* which completely deviates from other structures. The differences between these structures indicate that the gatekeeping loop is typically flexible in the apo state and can exhibit many conformations, but the order of flexibility differs between species, potentially caused by intrinsic dynamic properties.

**FIG. 2.**
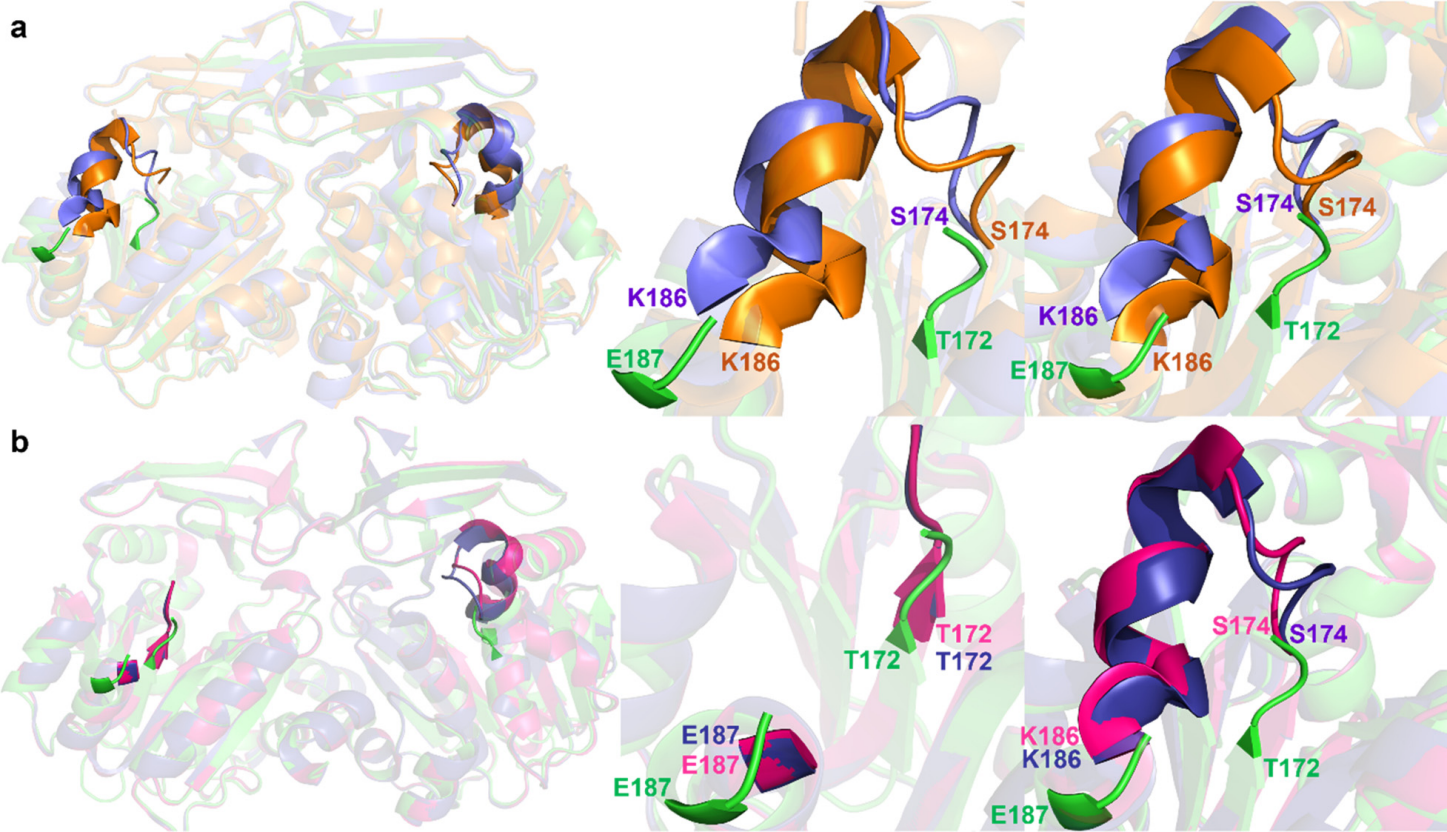
Structural comparison of the gatekeeping loop of the apo human SPDS structure with human ligand-bound SPDS structures. Superposition of apo human SPDS in green residues with (a) SPDS-MTA complex (2O05) in purple and SPDS-MTA-PUT complex (2O06) in orange, (b) SPDS-MTA-SPD complex (2O07) in deep blue and SPDS-dcAdoMet complex (2O0L) in pink. Left: Overall structure; center: chain A; right: chain B.

Comparisons of the structure of apo human SPDS to human ligand-bound SPDS structures revealed the extremes in the order/disorder of the gatekeeping loop (Fig. 2). Superposition of the apo form to SPDS-MTA (PDB 2O05) and SPDS-MTA-PUT (PDB 2O06) complexes shows the ligand-bound structures are ordered in both chains with complete residues and the presence of the short alpha helix in the gatekeeping loop compared to the apo state that is highly disordered [Fig. 2(a)]. However, the structure of SPDS in complex with MTA was reported to still be flexible and was stabilized upon the binding of putrescine.^5,30^ Comparison of the apo SPDS structure with SPDS-MTA-SPD (PDB 2O07) and SPDS-dcAdoMet (PDB 2O0L) complexes shows an ordered loop in chain B of the ligand-bound structures. In contrast, chain A is similar to the apo form with the absence of residues from 176 to 186 [Fig. 2(b)]. It was predicted that the positioning of putrescine for the reaction is influenced by the stabilization of the gatekeeping loop, and the release of the product requires the loop to open.^5,15^ Previous work on the structure of SPDS with the dcAdoMet analog, dcSAH, also revealed a disordered gatekeeping loop (PDB 3RW9).^15^ The occupancy of the active site as well as the position of this loop affects the orientation and binding of putrescine.^5,15^ The complete disorder observed in apo human SPDS illustrates how important ligand interactions is to stabilize the gatekeeping loop and forming a distinct active site.

**FIG. 3.**
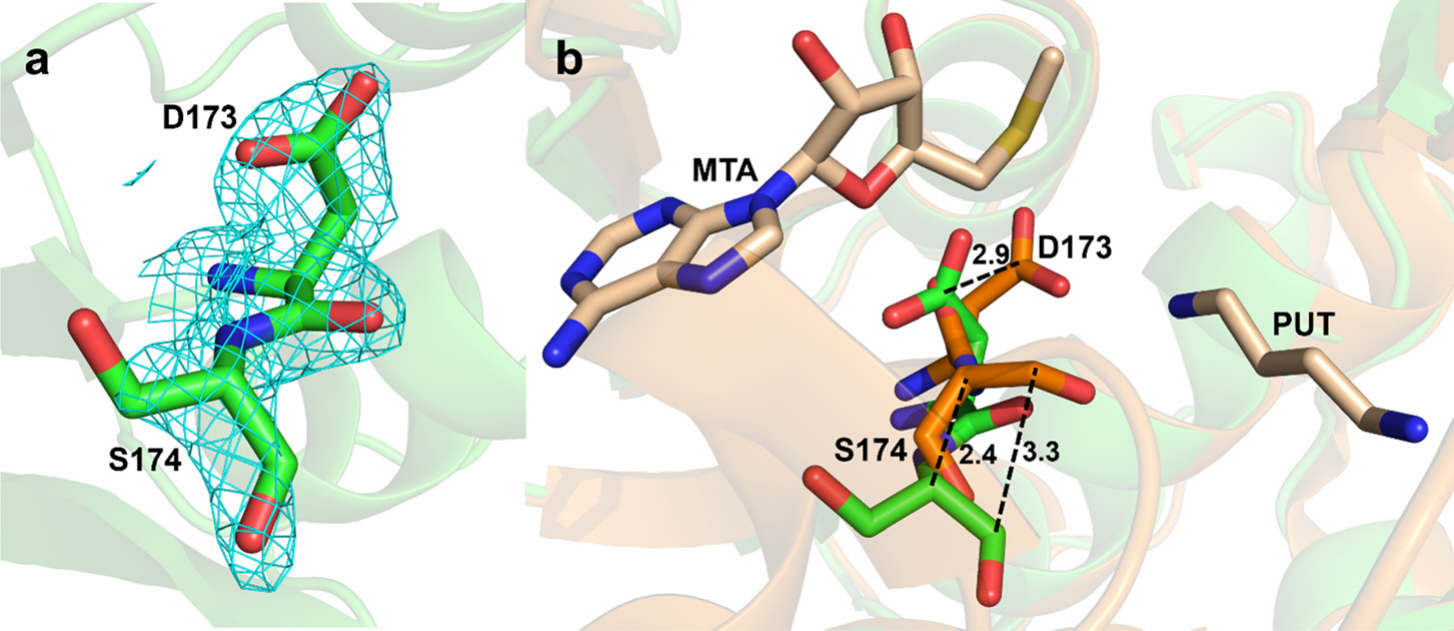
Comparison of Asp173 and Ser174 between apo human SPDS structure and SPDS-MTA-PUT complex. (a) Electron density map for residues D173 and S174 in chain A of apo human SPDS. Cyan density represents the 2mFo-DFc map contoured at RMSD of 1. The mFo-DFc maps are shown in Fig. S8(a) with orange density representing negative peaks contoured at RMSD of -3, and blue density representing positive peaks contoured at RMSD of 3. (b) Superposition of apo human SPDS structure and SPDS-MTA-PUT complex showing residues D173 and S174 at the active site.

**FIG. 4.**
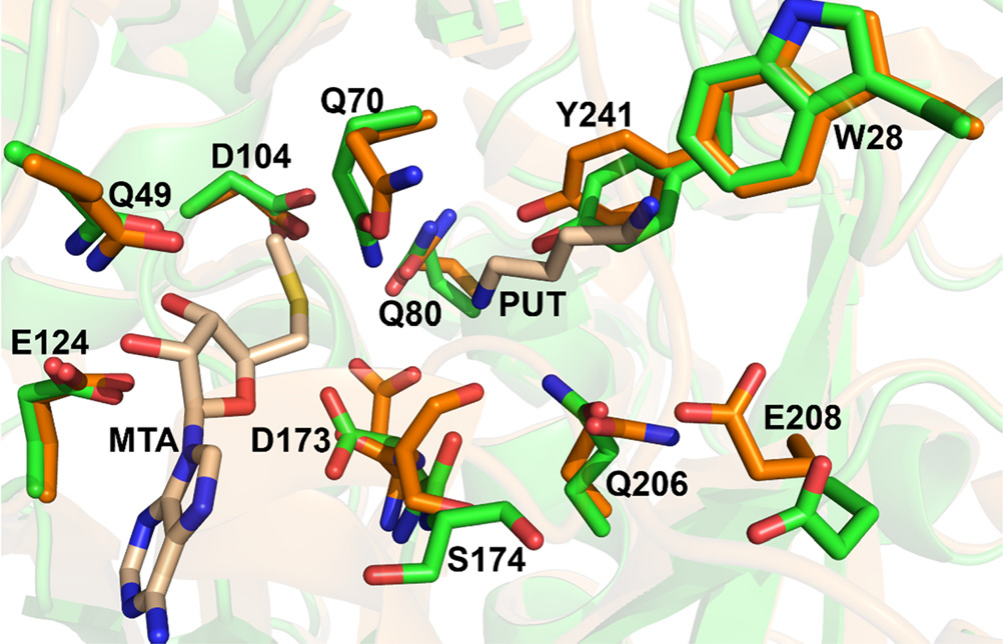
Active site residues comparison between apo human SPDS structure and SPDS-MTA-PUT complex. Apo SPDS structure in green and SPDS-MTA-PUT complex in orange.

In the apo structure, there is absence of electron density for the ligands, which is consistent with the ligand free state (Fig. S6). Comparison of the active site residues of SPDS-MTA-PUT complex (PDB 2O06) to the apo SPDS structure revealed differences in the position of some of the residues (Figs. 3 and 4). Asp173 and Ser174, important and conserved catalytic residues, showed the most striking changes. Asp173 deprotonates the N1 amine of putrescine, and the interaction is further strengthened by the interaction of the carbonyl group of Ser174 in preparation for the nucleophilic attack by the N1 amine of putrescine.^15,23,46^ These two residues are positioned differently with altered backbone and side chain in the apo structure compared to ligand-bound structures (Figs. 3 and S7), as it precedes the region of disorder and missing electron density in the gatekeeping loop of the apo structure. The residues are oriented away from the N1 amine of putrescine. The distance between the γ-carbons of Asp173 in chain A of the apo vs SPDS-MTA-PUT structure is 2.9 Å. The distances between the alpha carbons and the carbonyl carbons of Ser174 in the two structures are 2.4 and 3.3 Å, respectively. In chain B of the apo structure, Asp173 adopts this conformation but is a little disordered and Ser174 in this chain is more disordered than in chain A (Fig. S8). This conformation is not observed in apo structures from homologous species (Fig. S9), and it is also not seen in spermine synthase, which has a similar catalytic mechanism to SPDS. Plots from Ringer used to sample the electron density around the side chain dihedral angle of Asp173 in both A and B chains did not detect the ligand-bound conformation above the noise threshold, indicating that Asp173 is not sampling the ligand-bound conformation in the apo crystal (Fig. S10).

Some other residues at the active site are prepositioned regardless of ligand binding, for example, Trp28, which prevents the binding of longer amine substrates,^23^ retains the same position in the apo structure, and Glu124 and Gln49 that interact with the ribose of dcAdoMet. There are differences in other residues, notably, Tyr241, Gln80, Gln206, Gln70, and Glu208. Gln70, which is close to the site of the amino-propyl group transfer chemistry, is in a different rotameric state in the apo structure vs ligand-bound structures. Glu208 is oriented away from the active site compared to the ligand-bound state that is pointing toward the catalytic pocket. Tyr241 that interacts with the N1 amine of putrescine rotates more than 45°, and Gln80 that interacts with the aminopropyl group of dcAdoMet has a subtle shift in the side chain (Fig. 4). These residues in SPDS-MTA-PUT complex do not fit into the electron density of the apo structure, revealing the different conformation of some of these residues (Fig. S11). Thus, the binding of the ligand causes a shift in conformation of some of the active site residues for the transferase reaction to take place. Overall, the dcAdoMet binding site is more rigid, while the PUT binding site is more flexible. The largest and most interesting conformational changes take place in between the two, where the amino-propyl transfer occurs.

## CONCLUSION

This study presents the crystal structure of apo human SPDS at 1.95 Å resolution. In both chains A and B, residues 175–185 showed no interpretable density, reflecting the high mobility of the gatekeeping loop in the absence of ligand. Comparison of this structure with other homologous structures demonstrated that, although gatekeeping loop disorder is a common feature, there is variation in both the length and position of the missing residues, pointing to differences in the degree of disorder and flexibility across species.

**FIG. 5.**
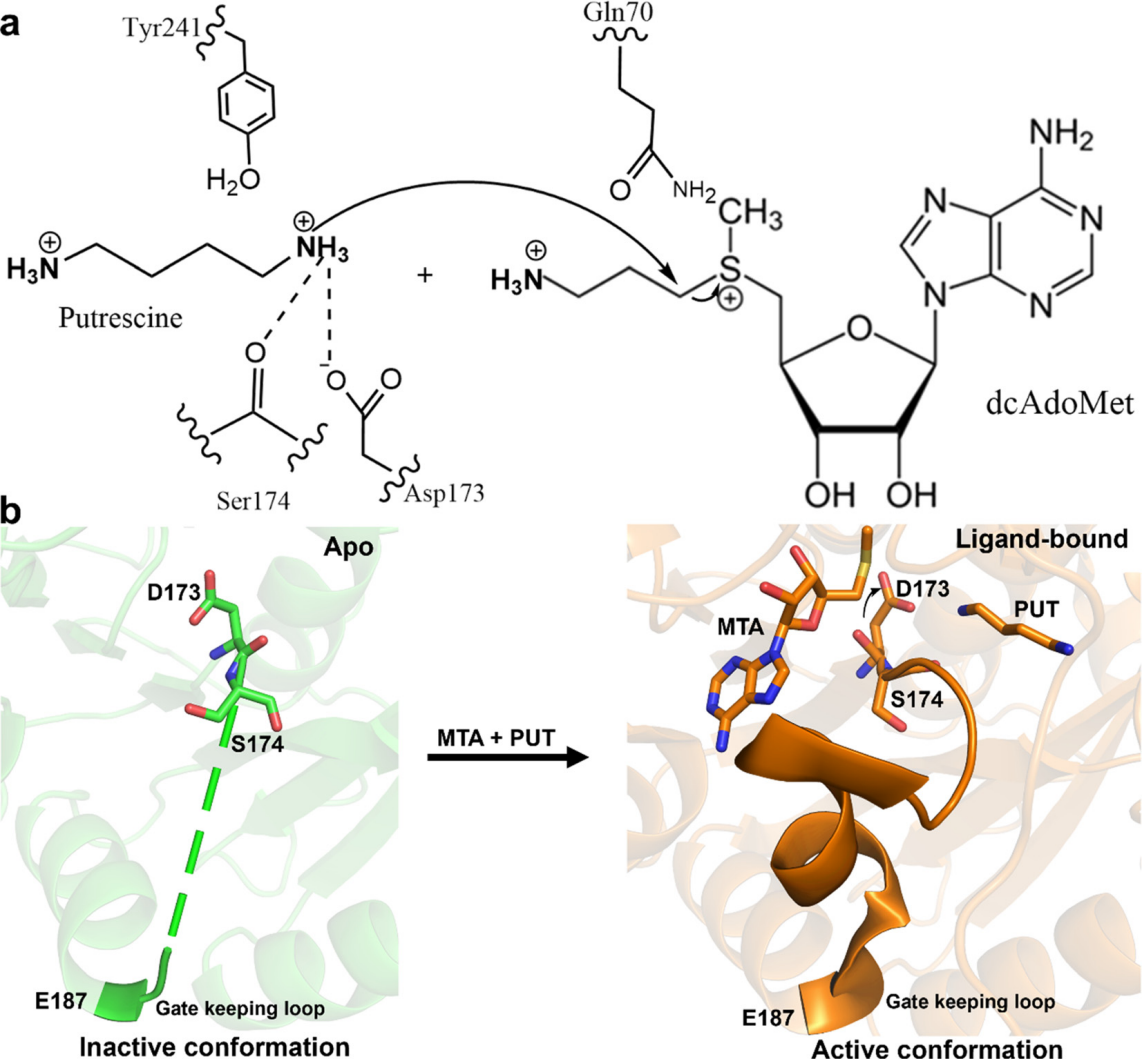
(a) Mechanism emphasizing catalytic residues undergoing conformational change. (b) Overview of ligand-induced conformational changes in human SPDS. Upon substrate binding, D173 and S174 change conformations and drive gatekeeping loop closure.

Structural comparison of the apo to ligand-bound structures revealed differences in the active site, notably the catalytic residues, D173 and S174, demonstrating conformational switches to facilitate substrate binding and catalysis. These motions are tied closely to the position of the active site gating loop, as these residues are located immediately prior to the loop in the sequence (Fig. 5). Additional active site residues in the aminopropyl transfer pocket and putrescine binding pocket also demonstrate altered conformations upon ligand binding. Optimization of crystallization and crystal extraction was also critical for obtaining high-quality diffraction, particularly given the crystal's needlelike morphology. These findings provide new structural details on human SPDS. This result reinforces the flexibility of the SPDS gating loop and active site. Future inhibitor design may benefit from targeting the apo active site instead of the enzyme–substrate complex surface.

## SUPPLEMENTARY MATERIAL

See supplementary material for Fig. S1: Representative images of mounted crystals at the beamline; Fig. S2: Diffraction pattern of the crystals looped at room temperature vs cold room; Fig. S3: Overall structure of apo human SPDS; Fig. S4: Protein sequence alignment of SPDS from different organisms generated using ESPript 3.0; Fig. S5: Gatekeeping loop comparisons among homologues; Fig. S6: Active site of apo human SPDS structure; Fig. S7: Superposition of apo human SPDS and human ligand-bound structures showing conserved Asp173 and Ser174 residues; Fig. S8: Active site residues, Asp173 and Ser174 of apo human SPDS; Fig. S9: Comparison of Asp173 and Ser174 between apo human SPDS structure and homologous structures; Fig. S10: Ringer plots of Asp173 residue comparing apoSPDS and SPDSMTAPUT (2O06); Fig. S11: The 2Fo-Fc map (blue) for the active site residues in the apo human SPDS structure.

## Data Availability

Apo human SPDS was deposited in the protein data bank with PDB ID 9Q41. It contains unmerged data, scaled and merged data, atomic coordinates, and the corresponding electron density map from final refinement. Raw diffraction images, including the master.h5 file, were deposited on the sbgrid.org data bank with dataset number 1194 (doi: 10.15785/SBGRID/1194).
